# COVID-19 myopericarditis with cardiac tamponade in the absence of respiratory symptoms: a case report

**DOI:** 10.1186/s13256-020-02618-z

**Published:** 2021-01-25

**Authors:** Lauren Cairns, Yazeed Abed El Khaleq, Will Storrar, Michaela Scheuermann-Freestone

**Affiliations:** Basingstoke and North Hampshire Hospital, Hampshire Hospitals Foundation Trust, Hampshire, United Kingdom

**Keywords:** COVID-19, Myocardial, Cardiac complications, Cardiac tamponade, Case report

## Abstract

**Background:**

Previous reports have shown various cardiac complications to be associated with COVID-19 including: myocardial infarction, microembolic complications, myocardial injury, arrythmia, heart failure, coronary vasospasm, non-ischemic cardiomyopathy, stress (Takotsubo) cardiomyopathy, pericarditis and myocarditis. These COVID-19 cardiac complications were associated with respiratory symptoms. However, our case illustrates that COVID-19 myopericarditis with cardiac tamponade can present without respiratory symptoms.

**Case presentation:**

A 58-year-old Caucasian British woman was admitted with fever, diarrhoea and vomiting. She developed cardiogenic shock and Transthoracic echocardiogram (TTE) found a pericardial effusion with evidence of cardiac tamponade. A nasopharyngeal swab showed a COVID-19 positive result, despite no respiratory symptoms on presentation. A pericardial drain was inserted and vasopressor support required on intensive treatment unit (ITU). The drain was removed as she improved, an antibiotic course was given and she was discharged on day 12.

**Conclusions:**

Our case demonstrates that patients without respiratory symptoms could have COVID-19 and develop cardiac complications. These findings can aid timely diagnosis of potentially life-threatening COVID-19 myopericarditis with cardiac tamponade.

## Background

The ongoing coronavirus (COVID-19) outbreak started with the first case in December 2019 in Wuhan, China. It is caused by severe acute respiratory syndrome coronavirus 2 (SARS-CoV-2) and predominantly affects the respiratory system. However, there have been increasing reports of cardiac complications resulting from COVID-19 infections. We present a 58-year-old woman admitted with cardiac tamponade secondary to COVID-19 without respiratory signs or symptoms.

## Case presentation

**Fig. 1.**
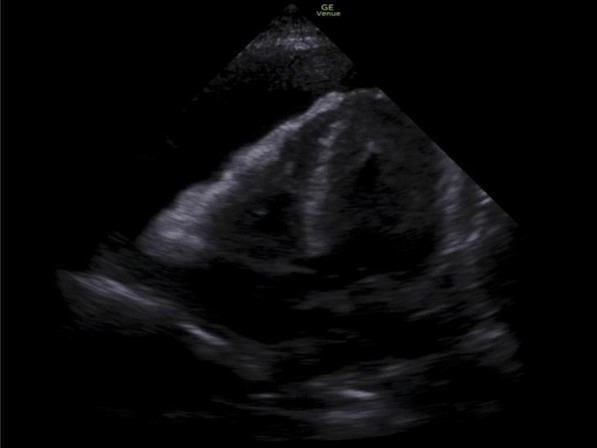
TTE showing the pericardial effusion and cardiac tamponade

**Fig. 2.**
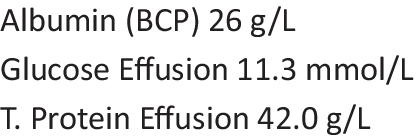
Analysis of pericardial fluid

A 58-year-old Caucasian British female patient presented with 10 day history of fever and 7 day history of diarrhoea, vomiting and poor oral intake, on a background of type 2 Diabetes and Hypertension. On admission she was hypotensive with blood pressure 85/45, respiratory rate 18, oxygen saturations 96% on air, heart rate 91 and temperature 34.7 °C. On examination, she had raised jugular venous pressure (JVP), pulsus paradoxus and generalised abdominal tenderness. Transthoracic echocardiogram (TTE) showed 1.5 cm pericardial effusion initially, over 7 hours the effusion progressed to 3–4 cm with evidence of cardiac tamponade in intensive treatment unit (ITU) (Fig. 1). A pericardial drain was inserted, 500 ml of serous fluid aspirated and vasopressor support required (report for pericardial fluid analysis shown in Fig. 2). Her cardiovascular status improved following pericardiocentesis and the drain remained in situ for 2 days.

**Fig. 3.**
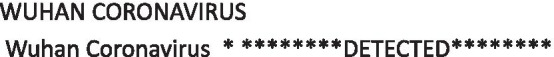
COVID-19 nasopharyngeal swab result

**Fig. 4.**
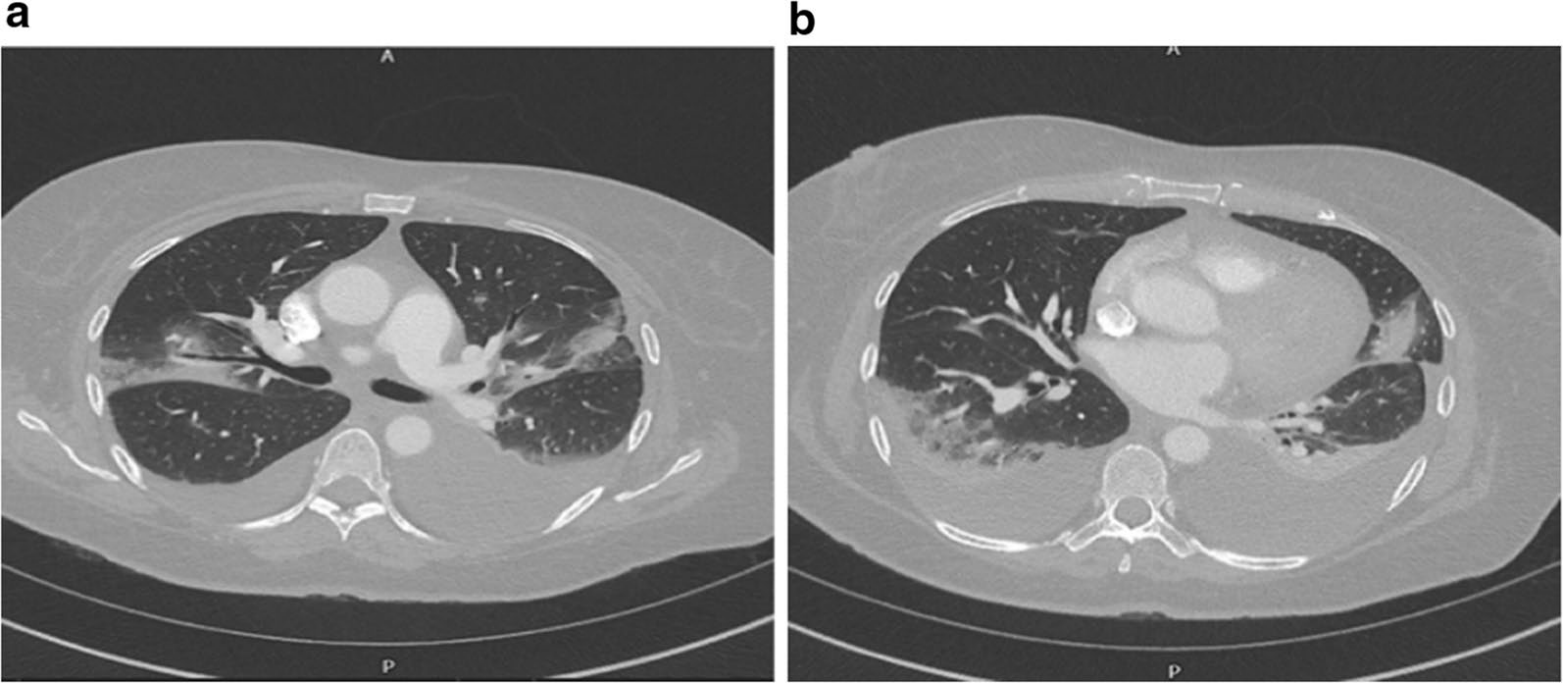
**a** Computed tomography chest scan showing bilateral consolidation. **b** Computed tomography chest scan showing bilateral consildation and large bilateral pleural effusions

**Fig. 5.**
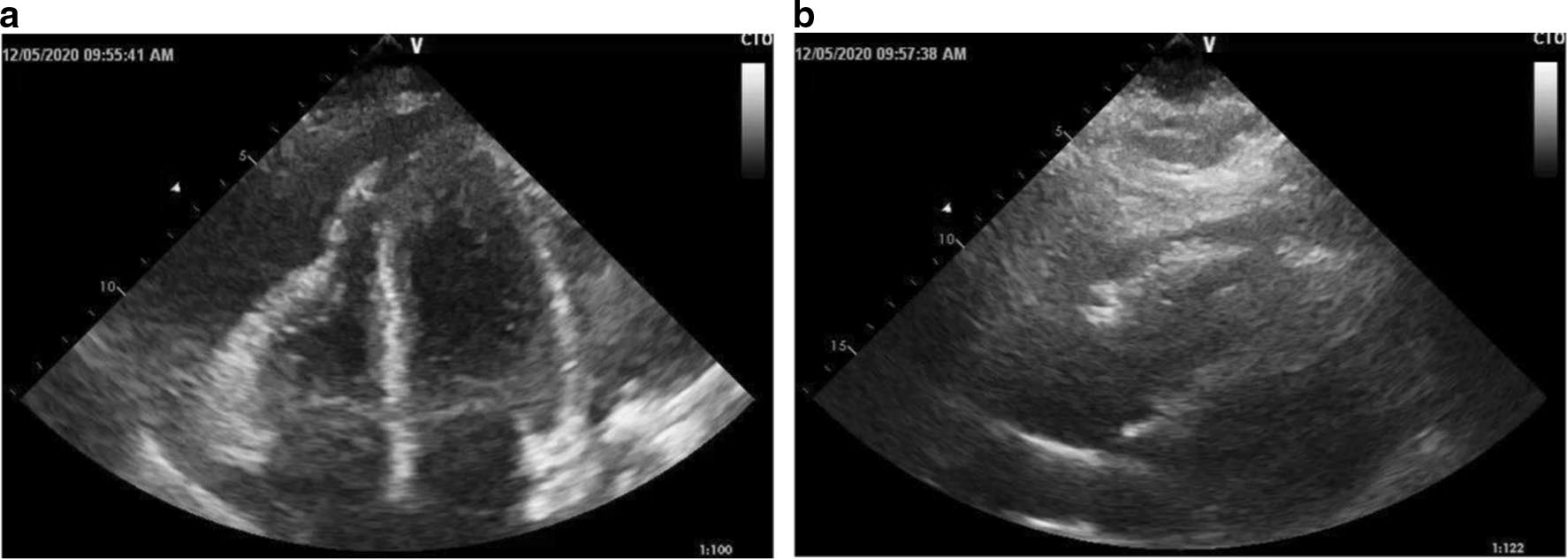
Repeat transthoracic echocardiogram with smaller pericardial effusion. **a** parasternal window, **b** subcostal window

A nasopharyngeal swab was taken to ascertain her COVID-19 status, which showed a positive result (Fig. 3). Computed tomography (CT) chest scan findings determined bilateral chest consolidation indicative of COVID-19 (Fig. 4). Bloods showed raised inflammatory markers, ferritin and lactate dehydrogenase. High sensitivity troponin was 388.8 ng/L (0–4.9) on admission, increasing to 3532.9 ng/L the next day. Atypical pneumonia, lymphoma and myeloma screens were all negative. She was treated with intravenous Amoxicillin and oral Doxycycline initially; these were escalated to Piperacillin/Tazobactam following an increase in inflammatory markers and temperature spike. Repeat CT chest scan showed a 1.2 cm in depth recurrent pericardial effusion with some progressive lung changes. She was commenced on furosemide due to bilateral pitting oedema. Repeat TTE showed a smaller global layer of pericardial effusion (1.2–1.4 cm) with no evidence of haemodynamic compromise (Fig. 5). She improved clinically and biochemically, Antibiotics were stopped and she was discharged on day 12. Following discharge, repeat chest x ray and TTE were arranged with outpatient respiratory and cardiology follow up.

## Discussion

Fever and dry cough were the first reported common symptoms in COVID-19 positive patients [1]. Studies have demonstrated the following cardiac complications to be associated with COVID-19: myocardial infarction, microembolic complications, myocardial injury, arrythmia, heart failure, coronary vasospasm, non-ischemic cardiomyopathy, stress cardiomyopathy, pericarditis and myocarditis [2, 3]. There is little known about myocarditis as a COVID-19 complication. Viral infections including influenza, are the most common infectious cause of myocarditis [4]. Cardiac tamponade has rarely been reported as a COVID-19 complication.

There are multiple case reports illustrating myopericarditis in COVID-19 patients in conjunction or after the onset of respiratory symptoms. Two of the cases report no previous medical history [3, 4]. Past medical history in the other cases include non-ischemic cardiomyopathy [5] and previous myopericarditis [6]. It seems plausible that previous cardiovascular co-morbidity could increase risk of COVID-19 myopericarditis, although further studies would be required [7, 8].

Size of pericardial effusion reported ranges from 1.1 to 2 cm, smaller compared to the pericardial effusion in our case (3–4 cm). Three case reports diagnosed cardiac tamponade with TTE requiring pericardiocentesis. These cases recorded pericardial fluid drained as 300 ml serous fluid [3], 540 ml serous fluid [6] and 800 ml of exudative bloody fluid [5]. Where possible the PCR of the fluid was tested and found to be COVID-19 negative, supporting the findings in our case [3, 5, 6]. Takotsubo cardiomyopathy was identified post pericardiocentesis in the third case [5].

Troponin was raised in all expect one case report. As troponin can be raised with pneumonia, myocardial damage can be differentiated with TTE or cardiac MRI if necessary [2]. ECG findings were recorded as ST elevation or nonspecific ST changes [2, 5, 6].

Similar to our case, two patients were prescribed heart failure medication such as Furosemide [2, 5]. Although, antibiotics were not documented to have been given in the other reported cases. The main therapies to treat myopericarditis include non-steroidal anti-inflammatories and glucocorticoids [3]. Four cases mentioned which medications were used. Three received glucocorticoids, two were prescribed colchicine in addition. Three also received trial COVID therapies including hydroxychloroquine and antiretrovirals, however these therapies have not been validated. Moreover, no medications have currently been recommended to treat COVID-19 myopericarditis [2–5].

Although there is no clear mechanism for the pathogenesis of cardiac involvement, various methods have been proposed [4, 5]. SARS-CoV2 could reflect the dissemination process of the virus through blood or lymphatics of the respiratory tract [5]. Conversely, an inflammatory response, similar to other viruses, could be triggered resulting in pericarditis and pericardial effusion [4].

## Conclusion

Our case highlights that COVID-19 myopericarditis can be complicated by cardiac tamponade and can present without respiratory symptoms. Further studies as the pandemic progresses will be required to develop better understanding of the pathogenesis, presentation and specific treatment for COVID-19 myopericarditis.

## Data Availability

The data for this case report is located at Basingstoke and North Hampshire Hospital.
